# Heterogeneity in disparities by income in cardiovascular risk factors across 209 US metropolitan areas

**DOI:** 10.1016/j.pmedr.2024.102908

**Published:** 2024-10-19

**Authors:** John Kundrick, Heather Rollins, Pricila Mullachery, Asma Sharaf, Alina Schnake-Mahl, Ana V. Diez Roux, Usama Bilal

**Affiliations:** a Department of Health Management and Policy, Drexel Dornsife School of Public Health, Philadelphia, PA, USA; b Drexel University College of Medicine, Philadelphia, PA, USA; c Department of Medicine, University of Pittsburgh Medical Center, Pittsburgh, PA, USA; d Urban Health Collaborative, Drexel Dornsife School of Public Health, Philadelphia, PA, USA; e Department of Health Services Administration and Policy, College of Public Health, Temple University, USA; f Department of Epidemiology and Biostatistics, Drexel Dornsife School of Public Health, Philadelphia, PA, USA

**Keywords:** Disparities, Income, Urban health, Cardiovascular disease, Obesity, Diabetes, Hypertension, Smoking, Sedentarism

## Abstract

**Objective:**

The United States has a lower life expectancy and wider income inequality than its similarly developed counterparts, and disparities continue to widen. The objective of our study is to examine the heterogeneity of disparities by income in cardiovascular disease (CVD) risk factors among U.S. metropolitan areas.

**Methods:**

Data was obtained from the Behavioral Risk Factor Surveillance System for 2012–2019. We used self-reported data for respondent characteristics and for CVD risk factors/prevalence, and on metropolitan-level demographic and socioeconomic characteristics. We computed the relative index of inequality (RII) for each outcome using a multilevel Poisson model, sequentially adjusted for age, sex, and race/ethnicity with a random slope for income. We also included interactions between income and the metropolitan-level variables.

**Results:**

Our sample included 1.4 million participants from 209 metropolitan areas. All CVD risk factors and CVD demonstrated income-related disparities. There were no clear regional patterns for risk factors, though seven of the top 10 large metropolitan areas with the widest disparities in CVD prevalence were in the South. Improved socioeconomic conditions were associated with wider disparities in the five risk factors, and contextual variables explained almost half of the variability in income disparities in smoking, sedentarism, and obesity, even after adjusting for age, sex, and race/ethnicity.

**Conclusions:**

This study found that CVD risk factors and prevalence in U.S. metropolitan areas have heterogeneous income disparities, especially in advantaged metropolitan areas. Further studies with improved data collection may shed more light into potential drivers of income-based disparities in cardiovascular risk.

## Highlights

• Income-related disparities in cardiovascular risk factors and prevalence are heterogenous among US cities.
• Smoking demonstrated the greatest heterogeneity in magnitude of disparities.
• Improved socioeconomic conditions were associated with wider disparities.

## 1 Introduction

The United States (US) has a lower life expectancy than similarly developed countries, ranking 50th in life expectancy worldwide in 2018 (Harper et al., 2021). Furthermore, life expectancy declined for the first time in the 21st century in 2015 and continued declining the two subsequent years, causing an overall stagnation in life expectancy for the decade (2010–2019) (Harper et al., 2021, Case and Deaton, 2015). These changes in life expectancy have been driven by an increase in drug- and alcohol-related deaths (Harper et al., 2021), and a stagnation in cardiovascular disease (CVD) mortality, after years of improvement (Case and Deaton, 2015, Mehta et al., 2020). These worrisome developments obscure other intra-country patterns, such as a widening of disparities in life expectancy between states (Woolf and Schoomaker, 2019, Montez et al., 2020) and counties (Dwyer-Lindgren et al., 2017), a stagnation of previously declining inequities by race/ethnicity (Bilal and Diez-Roux, 2018), and a concentration of increases in mortality among adults with lower levels of education, regardless of race/ethnicity (Case and Deaton, 2021).

Higher income individuals, families, and households experience lower morbidity and mortality than their low-income counterparts, (Dwyer-Lindgren et al., 2017, Hunt et al., 2015, Lynch et al., 1998, Spatz et al., 2016, Gebreab et al., 2015, Rosengren et al., 2019, Polyakova and Hua, 2019, Zhu et al., 2021, Kibria et al., 2021, Montez and Berkman, 2014, Hines et al., 2014, Ross et al., 2000, Lynch et al., 2005, Kahn et al., 1998, Patel et al., 2016, Sanmartin et al., 2003, Ross et al., 2005, Boykin et al., 2011, Chetty et al., 2016, Mackenbach et al., 2008, Odutayo et al., 2017) particularly in the US, where income inequality is greater and mortality disparities are wider, when compared to other developed countries. (Ross et al., 2000, Sanmartin et al., 2003) Importantly, these disparities in mortality and in life expectancy by income continue to widen. (Chetty et al., 2016) Furthermore, income inequality itself, beyond the role of individual income, is important in determining mortality in the US. Low income people living in areas with wider income inequality have higher mortality (Lynch et al., 1998), and there is a strong correlation between income inequality with mortality in US metropolitan areas. (Sanmartin et al., 2003) On the other hand, in Canadian metropolitan areas, income inequality is narrower and mortality rates are lower, and there is not a significant association between income inequality and mortality. (Sanmartin et al., 2003).

Better understanding of the magnitudes of inequities by income in CVD and the factors associated with larger or smaller income inequities can help to inform policies to reduce CVD mortality levels and mitigate disparities in CVD mortality. While prior research has focused on individual-level CVD disparities, and determinants or modifiers of disparities, there is a dearth of research examining the magnitude and drivers of income-related disparities in CVD and its risk factors across metropolitan areas in the US. Assessing the magnitude of income disparities in CVD and factors associated with wider (or narrower) disparities can help to inform potential policy interventions to mitigate CVD disparities in the US, improving CVD risk, morbidity, and mortality for all. Our objective was to examine heterogeneity in disparities by income in CVD risk factors (obesity, smoking, diabetes, hypertension, and sedentarism) and CVD prevalence within US metropolitan areas from 2012 to 2019, and to explore predictors of the magnitude of disparities at the metropolitan level.

## 2 Methods

### 2.1 Study Design

This is a multilevel study of individuals nested in metropolitan areas of the US. Data for this study was obtained from the 2012 through 2019 waves of the Selected Metropolitan/Micropolitan Area Risk Trends (SMART) version of the Behavioral Risk Factor Surveillance System (BRFSS), which includes data on individuals sampled in metropolitan statistical areas (a type of core-based statistical areas or CBSAs), with at least 500 respondents. More details on SMART/BRFSS are available in the Appendix. The final complete case sample with no missing outcomes, exposures, or covariates contained 1,419,441 individuals nested within 209 metropolitan areas. Projection weights provided with the data were rescaled to sum to the total sample size by year and metropolitan area.

### 2.2 Outcomes

We used six self-reported outcomes, including five CVD risk factors and prevalent CVD. These five risk factors were obesity, diabetes, hypertension, current smoking, and sedentarism. Obesity was measured as a body mass index (BMI) of equal to or greater than 30 kg/m^2^, using self-reported height and weight. We measured presence of diabetes, hypertension, and cardiovascular disease (heart attack, angina, coronary heart disease, or stroke), by a response of “yes” to the question about a medical professional indicating the patient had the respective disease. Smoking was measured by participants who responded that they had smoked at least 100 cigarettes in their lifetime and currently smoked at least some days. Respondents were determined to have a sedentary lifestyle if they reported doing no physical activity or exercise in the past 30 days other than their regular job. All six outcomes were operationalized as binary variables.

### 2.3 Exposure

The main explanatory variable was household income, henceforth income. Income was operationalized as a continuous variable with eight intervals (<$10 k, $10–15 k, $15–20 k, $20–25 k, $25–35 k, $35–50 k, $50–75 k, >=$75 k, inverted and rescaled between 0 (>$75 k) and 1 (<$10 k)). We inverted and rescaled income categories to facilitate the interpretation of coefficients (see statistical analysis section).

### 2.4 Covariates

For adjustment purposes, we also collected data on survey year, the respondent’s age (available as 5-year age groups and operationalized as a continuous variable using the midpoint of each category), sex (binary; male or female), and race/ethnicity (non-Hispanic White, non-Hispanic Black, Hispanic, and non-Hispanic other races). Age and sex were included as they are potential causes of the outcome and may be related to income. Race/ethnicity was considered a proxy of exposure to structural racism, which is a driver of both CVD (Powell-Wiley et al., 2022) and income (Bailey et al., 2017).

Additionally, we examined several area-level contextual factors that may affect the magnitude of the income-related disparities within each metropolitan area. We do not intend to test whether these factors are causally related to disparities, but rather to describe how disparities vary by commonly used measures of area-level features. Specifically, we collected data on the age distribution (% below age 18, % above age 64), demographic composition (% non-Hispanic Black, % Hispanic, % foreign-born), economic factors (% college educated, % below poverty, Gini Index (a measure of income inequality), and % unemployed), as these compositional variables vary by metropolitan area and we hypothesize they play a role in the heterogeneity in income-based disparities. Healthcare related factors were used as proxies of access to care (% uninsured and primary care physicians (PCPs) per population) for each metropolitan area. % uninsured was chosen given that insurance status may play a role in an individual’s utilization of and access to healthcare, which may put them at risk of CVD. PCPs per population was chosen as it was hypothesized that number of PCPs in each area may affect access to preventive care. Data sources included the 5-year American Community Survey, the Area Health Resource Files from the Health Resources & Services Administration, and labor force data by county from the Bureau of Labor Statistics, with all data corresponding to the years 2012 to 2019.

### 2.5 Statistical analysis

The main objective of this analysis was to explore the variability and predictors of income-related disparities in CVD risk factors and prevalence across US metropolitan areas. Appendix 1 contains more details on the modeling strategy. In summary, we used weighted robust multilevel Poisson models of individuals nested in metropolitan areas to estimate the magnitude of income disparities for each of the six binary risk factors through the relative index of inequality (RII) (Moreno-Betancur et al., 2015), which can be interpreted as the linearized prevalence ratio between the lowest and highest income levels. We used robust Poisson models rather than logistic in order to avoid dependence of odds ratios on the baseline prevalence, which may differ between risk factors and cities. All models were adjusted by year, and then sequentially by age, sex, and race/ethnicity. To explore contextual factors driving income-related disparities, we added, first in separate models and then jointly, the 11 metropolitan-level variables listed above. Finally, we assessed the change in variability in the income disparities across metropolitan areas by calculating the proportional change in variance (PCV) between a model with just income and year, and each subsequent model. We hypothesized that adjusting the model with individual level and contextual variables would reduce the variability of the income random slope across metropolitan areas.

This research is exempt from institutional review board review under 45 CF 46.104(d)(4)(i) (data is publicly available). Analysis was conducted using R v4.2.

## 3 Results

Table 1 displays characteristics of the 1.4 million survey respondents by income category in the study sample. The median number of respondents per metropolitan area was 3,133 (IQR 868 – 8,257). 22.8 % of participants were aged 18–39, 34.8 % were 40–59, and 42.4 % were 60 years of age or older. The majority of participants identified as non-Hispanic White (76.9 %), with 10.1 % identifying as non-Hispanic Black, 7.4 % identifying as Hispanic, and 5.6 % as non-Hispanic other. Females made up 54.4 % of respondents. The percentage of non-Hispanic whites and males tended to increase, and the percentage of all other races and females tended to decrease, as income increased. We found that 30 % of participants were classified as obese, 12 % had diabetes, 39 % had hypertension, 10 % were smokers, 23 % exhibited sedentary lifestyle, and 10 % reported having been diagnosed with a cardiovascular disease. Overall, the prevalence of each risk factor and CVD decreased as the income category increased.

**Table 1:** Characteristics and Demographics of Study Participants across 209 Metropolitan Areas of the United States from 2012 to 2019.

	**By Income Category (in thousands of US Dollars)**								
	**0**–**10**	**10**–**15**	**15**–**20**	**20**–**25**	**25**–**35**	**35**–**50**	**50**–**75**	**75+**	**Overall**
**N**	59,169	64,589	92,233	117,384	141,635	193,764	228,812	521,855	1.40 M
N per metropolitan area [Q1-Q3]	142 [49–––369]	149 [54–––405]	231 [73–––576]	280 [82–––747]	341 [99–––883]	457 [125–––1,213]	500 [146–––1,336]	857 [242–––2,668]	3,133 [868–––8,257]
**Age**									
18–39 (%)	28.2	19.1	23.7	24.0	23.1	23.3	23.3	21.8	22.8
40–59 (%)	37.5	29.7	26.6	24.8	23.8	26.9	32.9	45.6	34.8
60+ (%)	34.3	51.2	49.7	51.1	53.1	49.9	43.7	32.6	42.4
**Race/Ethnicity**									
Non-Hispanic White (%)	52.2	62.2	61.9	68.3	72.2	77.4	81.6	85.1	76.9
Non-Hispanic Black (%)	23.1	17.3	17.4	13.8	12.5	10.3	8.2	5.6	10.1
Hispanic (%)	16.0	13.9	14.1	11.9	9.7	7.1	5.1	3.9	7.4
Non-Hispanic Other (%)	8.6	6.6	6.6	6.0	5.6	5.2	5.0	5.4	5.6
**Sex**									
Male (%)	36.2	35.6	37.3	39.1	41.2	45.1	47.2	51.5	45.6
Female (%)	63.8	64.4	62.7	60.9	58.8	54.9	52.8	48.5	54.4
**Outcome**									
Obesity (%)	36.0	37.0	34.6	32.9	31.4	31.1	30.0	25.2	29.6
Diabetes (%)	19.3	22.1	19.2	17.0	15.3	13.2	11.1	7.3	12.3
Hypertension (%)	46.5	52.5	48.7	45.9	45.0	41.6	37.6	30.6	38.6
Smoking (%)	20.6	18.8	16.7	14.7	12.8	10.9	8.8	4.9	10.0
Sedentarism (%)	38.5	39.7	36.8	33.6	29.7	24.6	19.8	12.8	22.8
Cardiovascular Disease (%)	17.2	20.9	17.7	15.6	13.6	11.5	8.9	5.9	10.6

Description of the characteristics and prevalence of cardiovascular disease risk factors and prevalence of each participant sorted by income category. For all statistics (with the exception of “N per metropolitan area”), the unit of analysis is the individual. [Q1-Q3] represents the two extremes of the interquartile range (quartile 1 and 3). N = number of participants.

**Fig. 1:**
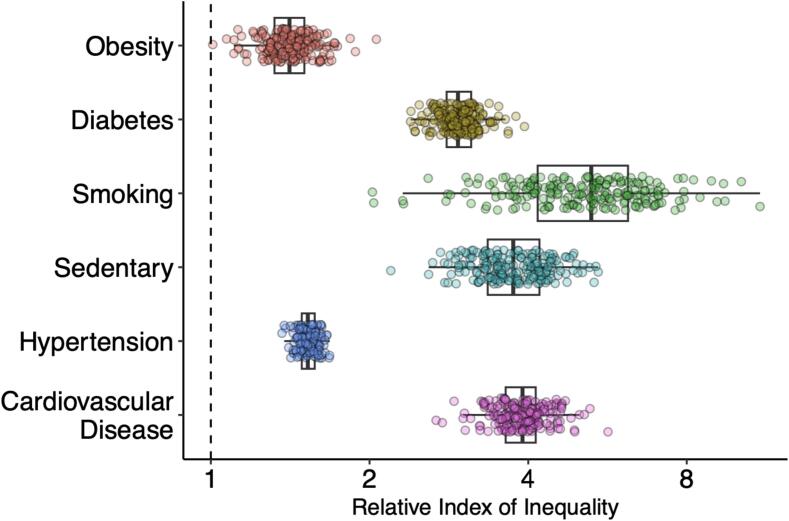
Income-based Inequalities for Each Risk Factor And Cardiovascular Disease Prevalence by Relative Index of Inequality, Adjusted for Age and Sex, in 209 Metropolitan Areas of the United States from 2012 to 2019. Footnote: Box and whisker plot of the Relative Index of Inequality (RII) of each risk factor for each core-based statistical area. Each point represents a metropolitan area. Higher RII is indicative of greater inequality. Greater variability in RII for each risk factor is indicative of greater heterogeneity in disparities for this risk factor across metropolitan areas. These were adjusted for age and sex.

Fig. 1 shows the RII of each individual risk factor and CVD for each metropolitan area, adjusted for age and sex (Appendix Fig. 1 show the RII and 95 % confidence interval for each metropolitan area and outcome). The RII is interpreted as a linearized ratio between the prevalences of each risk factor or CVD in the lowest vs highest income category in a given metropolitan area. Each point represents a metropolitan area and the further above 1, the wider the disparity for that cardiovascular risk factor or CVD. We observed an RII of greater than one in all categories and metropolitan areas, meaning that all risk factors and CVD demonstrated income-related disparities, with higher prevalence in lower income categories. In general, the average disparities were widest for smoking and narrowest for obesity. In addition, the heterogeneity in disparities by income for each risk factor or CVD is shown by the level of variability within each outcome in the figure, with heterogeneity metrics shown in Appendix Table 1. Smoking had the greatest heterogeneity of magnitude of disparities between metropolitan areas (coefficient of variation of 28.5 %), while hypertension had the lowest (coefficient of variation of 4.1 %). We also found, as shown in Appendix Fig. 2, that the RIIs by outcome were moderately correlated (ranging from 0.25 between smoking and obesity to 0.58 between sedentarism and obesity) for all outcomes, with the exception of CVD prevalence (correlations ranging from −0.07 to 0.13). Finally, Appendix Fig. 3 compares the RII using the global vs metropolitan-specific income distributions to calculate ridit scores, showing no changes to our inferences.

**Fig. 2:**
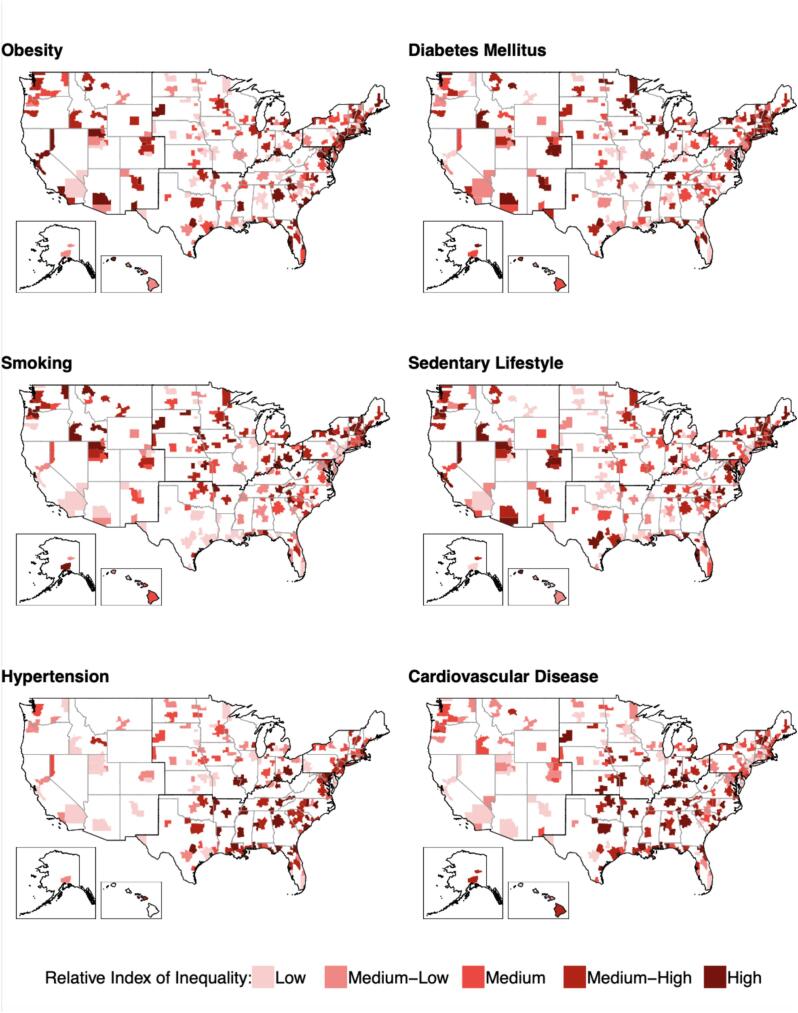
209 Metropolitan Areas of the United States Ranked by Relative Index of Inequality for Five Cardiovascular Disease Risk Factors and Prevalence, Adjusted for Age and Sex from 2012 to 2019. Footnote: Categories were created using outcome-specific quintiles. Metropolitan areas were placed in quintiles of the Relative Index of Inequality (RII) for each risk factor and color coded. Darker areas represent metropolitan areas with greater disparities in a given factor. These were adjusted for age and sex.

Fig. 2 maps the ranking of metropolitan area specific disparities, after adjusting for age and sex. Darker areas represent metropolitan areas with wider disparities. Darker lines separate four broader regions within the U.S. (Northeast, South, Midwest, and West). We did not observe any consistent spatial pattern across outcomes. However, some trends are apparent for each outcome individually. For obesity, diabetes mellitus, and sedentary lifestyle no region appears to contain significantly greater proportions of metropolitan areas with a high RII. Smoking appears to have wider disparities in metropolitan areas located in the northern states of the U.S. (Northeast, Midwest, and West regions). Hypertension appears to have wider disparities along the East Coast, with metropolitan areas with a high RII in the Northeast, South, and parts of the Midwest. For CVD prevalence, the West region appears to have a lower proportion of metropolitan areas with a high RII, while the South region has more metropolitan areas with a higher RII.

Table 2 ranks the ten metropolitan areas, with a population above 1 million, with the widest age, sex, and race-adjusted income-related disparities by risk factor or CVD. Several metropolitan areas are represented multiple times on these tables. These metropolitan areas show the widest disparities among all large metropolitan areas in the U.S. for multiple outcomes. For example, Denver (Colorado), Austin (Texas), and Raleigh (North Carolina) appear in the top 10 of widest disparities in four of the six outcomes, while Boston (Massachusetts), Hartford (Connecticut), and Minneapolis (Minnesota) appear three times. We found a few regional patterns. The South had seven of the top 10 metropolitan areas with the widest disparities for CVD prevalence, and five and four of the top 10 metropolitan areas for sedentarism and diabetes, respectively. On the other hand, the West had four of the most unequal metropolitan areas for obesity and smoking.

**Table 2:** United States Metropolitan Areas Over 1 Million Population with Widest Disparities by Relative Index of Inequality for Each Outcome Adjusted by Age, Sex, and Race from 2012 to 2019.

**Outcome**	**Rank**	**Metropolitan Area**	**US Region**	**Population (millions)**	**RII (95 % CI)**
Obesity	1	San Jose-Sunnyvale-Santa Clara	West	1.94	1.65 (1.35, 2.01)
	2	Boston-Cambridge-Newton	Northeast	4.73	1.60 (1.47, 1.75)
	3	San Francisco-Oakland-Berkeley	West	4.58	1.51 (1.29, 1.77)
	4	Hartford-East Hartford-Middletown	Northeast	1.21	1.50 (1.34, 1.68)
	5	Sacramento-Roseville-Folsom	West	2.24	1.49 (1.27, 1.76)
	6	Grand Rapids-Kentwood	Midwest	1.03	1.49 (1.27, 1.75)
	7	Raleigh-Cary	South	1.24	1.49 (1.24, 1.79)
	8	Austin-Round Rock-Georgetown	South	1.94	1.48 (1.29, 1.70)
	9	Los Angeles-Long Beach-Anaheim	West	13.19	1.47 (1.31, 1.65)
	10	Jacksonville	South	1.42	1.45 (1.26, 1.67)
Diabetes	1	Austin-Round Rock-Georgetown	South	1.94	3.29 (2.63, 4.11)
	2	Hartford-East Hartford-Middletown	Northeast	1.21	2.85 (2.38, 3.41)
	3	Nashville-Davidson--Murfreesboro--Franklin	South	1.79	2.82 (2.19, 3.62)
	4	Denver-Aurora-Lakewood	West	2.75	2.79 (2.34, 3.33)
	5	Orlando-Kissimmee-Sanford	South	2.33	2.79 (2.22, 3.51)
	6	Minneapolis-St. Paul-Bloomington	Midwest	3.49	2.76 (2.38, 3.19)
	7	Raleigh-Cary	South	1.24	2.71 (2.06, 3.57)
	8	Rochester	Northeast	1.08	2.70 (2.11, 3.47)
	9	Providence-Warwick	Northeast	1.61	2.69 (2.33, 3.11)
	10	Phoenix-Mesa-Chandler	West	4.49	2.69 (2.28, 3.17)
Hypertension	1	Boston-Cambridge-Newton	Northeast	4.73	1.55 (1.42, 1.70)
	2	Austin-Round Rock-Georgetown	South	1.94	1.52 (1.36, 1.70)
	3	St. Louis	Midwest	2.80	1.51 (1.36, 1.68)
	4	Hartford-East Hartford-Middletown	Northeast	1.21	1.50 (1.35, 1.66)
	5	Grand Rapids-Kentwood	Midwest	1.03	1.49 (1.33, 1.67)
	6	Minneapolis-St. Paul-Bloomington	Midwest	3.49	1.49 (1.36, 1.62)
	7	Seattle-Tacoma-Bellevue	West	3.67	1.48 (1.34, 1.64)
	8	Providence-Warwick	Northeast	1.61	1.48 (1.35, 1.62)
	9	Los Angeles-Long Beach-Anaheim	West	13.19	1.48 (1.33, 1.64)
	10	Denver-Aurora-Lakewood	West	2.75	1.48 (1.34, 1.63)
Smoking	1	Portland-Vancouver-Hillsboro	West	2.35	8.08 (6.56, 9.95)
	2	Orlando-Kissimmee-Sanford	South	2.33	7.94 (5.82, 10.84)
	3	Boston-Cambridge-Newton	Northeast	4.73	7.68 (6.61, 8.92)
	4	Cincinnati	Midwest	2.15	7.67 (6.19, 9.50)
	5	Columbus	Midwest	2.00	7.63 (6.08, 9.57)
	6	Seattle-Tacoma-Bellevue	West	3.67	7.42 (6.13, 8.99)
	7	Denver-Aurora-Lakewood	West	2.75	7.35 (6.17, 8.76)
	8	Rochester	Northeast	1.08	7.35 (5.47, 9.87)
	9	Minneapolis-St. Paul-Bloomington	Midwest	3.49	7.33 (6.33, 8.50)
	10	Salt Lake City	West	1.15	6.94 (5.61, 8.60)
Sedentarism	1	Denver-Aurora-Lakewood	West	2.75	4.79 (4.21, 5.46)
	2	Milwaukee-Waukesha	Midwest	1.57	4.74 (3.90, 5.76)
	3	Raleigh-Cary	South	1.24	4.40 (3.42, 5.65)
	4	Tucson	West	1.00	4.36 (3.10, 6.15)
	5	Washington-Arlington-Alexandria	South	6.01	4.30 (3.92, 4.71)
	6	Indianapolis-Carmel-Anderson	Midwest	1.97	4.14 (3.61, 4.76)
	7	San Jose-Sunnyvale-Santa Clara	West	1.94	4.14 (3.21, 5.34)
	8	San Antonio-New Braunfels	South	2.33	4.14 (3.37, 5.08)
	9	Charlotte-Concord-Gastonia	South	2.38	4.10 (3.45, 4.87)
	10	Tampa-St. Petersburg-Clearwater	South	2.93	4.08 (3.41, 4.88)
Cardiovascular Disease	1	Kansas City	Midwest	2.07	5.24 (4.45, 6.17)
	2	Cincinnati	Midwest	2.15	4.96 (4.01, 6.13)
	3	Raleigh-Cary	South	1.24	4.55 (3.47, 5.96)
	4	Atlanta-Sandy Springs-Alpharetta	South	5.61	4.55 (3.73, 5.54)
	5	Jacksonville	South	1.42	4.54 (3.64, 5.67)
	6	Indianapolis-Carmel-Anderson	Midwest	1.97	4.52 (3.72, 5.50)
	7	Nashville-Davidson--Murfreesboro--Franklin	South	1.79	4.39 (3.44, 5.59)
	8	Dallas-Fort Worth-Arlington	South	6.96	4.32 (3.46, 5.41)
	9	Houston-The Woodlands-Sugar Land	South	6.48	4.26 (3.43, 5.30)
	10	Austin-Round Rock-Georgetown	South	1.94	4.24 (3.34, 5.39)

Depiction of the 10 metropolitan areas with at least 1 million population with the greatest disparities by the relative index of inequality (RII) for each cardiovascular disease risk factor or prevalence. These were adjusted for age, sex, and race. US: United States.

**Fig. 3:**
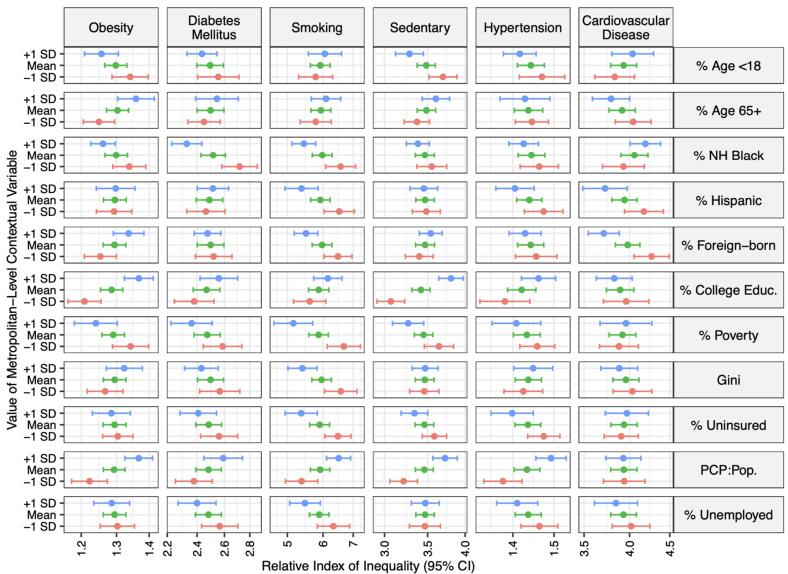
Relative Index of Inequality, Adjusted for Age, Sex, and Race/Ethnicity, in 209 Metropolitan Areas of the United States at the Average, +1 Standard Deviation, and –1 Standard Deviation of Metropolitan-Level Contextual Variables from 2012 to 2019. Footnote: Depicts the relative index of inequality (RII) (and 95 % confidence interval) of each contextual variable that was studied. The green bar represents the average metropolitan area level of each variable, the blue bar represents metropolitan areas with + 1 SD of each variable and the red bar represents metropolitan areas with −1 SD of each variable. Given this, contextual variables where the blue bar is to the right of the green bar and red bar is to the left of the green bar demonstrate greater disparities with greater values of that variable and vice versa. These were adjusted for age, sex, and race/ethnicity. NH: non-Hispanic, PCP pop: primary care physicians per capita. (For interpretation of the references to color in this figure legend, the reader is referred to the web version of this article.)

Fig. 3 shows the RII for each outcome in metropolitan areas at the average levels (mean) of each contextual variable, and in metropolitan areas at −1 and + 1 standard deviations (SD) for each variable, and Appendix Table 2 shows the p-values for the interaction coefficients between income and each variable. Each variable was included separately in a model adjusted for age, sex, and race/ethnicity. The green bar represents the RII (95 % CI) for a metropolitan area with average levels of each contextual variable, while the blue and red bars represent the RII (95 % CI) in metropolitan areas with + 1 SD and −1SD of each contextual variable. For the composition variables (% age < 18, % age 65+, % non-Hispanic Black, % Hispanic, and % foreign-born), the higher the percentage of minorities, the narrower the disparities for most risk factors overall, with a few exceptions, while for age, the older the metropolitan area the wider the disparities in obesity and CVD. For the socioeconomic variables (% college educated, % poverty, and % unemployed), the greater the percentage of higher socioeconomic status individuals in the population, the wider the disparities are overall, especially for the five risk factors. For the healthcare-related factors (% uninsured and PCPs per population), the less percentage uninsured and the more PCPs per population, the wider the disparities.

**Fig. 4:**
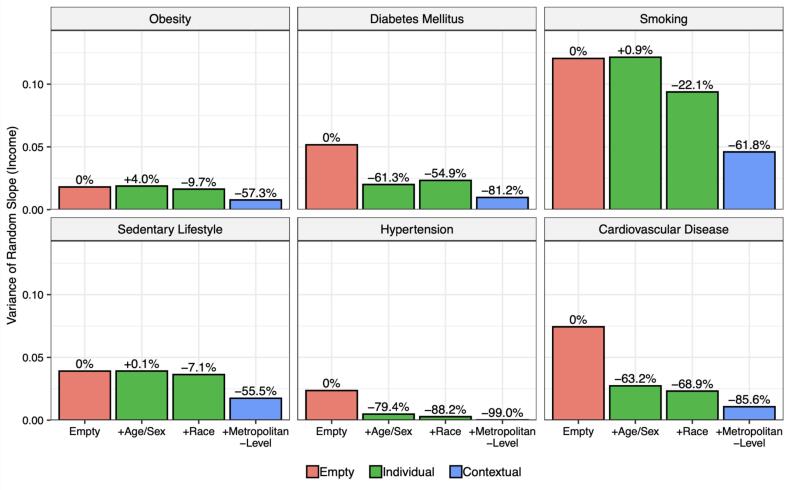
Magnitude of Heterogeneity in Income-Based Inequalities in Cardiovascular Risk Factors and Prevalence in Four Sequentially Adjusted Models across 209 Metropolitan Areas of the United States from 2012 to 2019. Footnote: Depicts the amount of heterogeneity in each risk factor that can be accounted for by sequentially adjusting for each variable. The pink bar represents heterogeneity before adjustment (empty or unadjusted model). The green bars represent the change in heterogeneity after adjusting for individual variables: age, sex, and (for the second green bar) race. The blue bar represents the heterogeneity after adjusting for the contextual variables. The closer to −100%, the more heterogeneity can be described by a given variable. (For interpretation of the references to color in this figure legend, the reader is referred to the web version of this article.)

Fig. 4 shows the variance of the income random slope, representing the variability in the RIIs across metropolitan areas, along with the % of this heterogeneity explained by sequentially adjusting for age and sex, race/ethnicity (individual variables), and all contextual variables shown in Fig. 3. In all six risk factors, all individual and contextual factors combined (blue bar) explained 55–99 % of the heterogeneity. This means that the majority of the variability between metropolitan areas can be explained by these variables. However, the degree to which this heterogeneity in disparities is explained by individual versus contextual variables varies by risk factor. Heterogeneity in obesity, smoking, and sedentary lifestyle is explained more by the contextual variables, consistent with the wider heterogeneity in disparities shown in Fig. 1; for these outcomes, contextual variables together explained 47.6 %, 39.7 %, and 48.4 % of the variability in disparities beyond what individual-level variables (age, sex, and race/ethnicity) explained, respectively. On the other hand, heterogeneity in disparities in diabetes mellitus, hypertension, and cardiovascular disease was mostly explained by individual level variables, with 54.9 %, 88.2 %, and 68.9 % of the heterogeneity in disparities for these outcomes explained by age, sex, and race/ethnicity, and only 26.3 %, 10.8 %, and 16.7 % explained by contextual variables, respectively.

## 4 Discussion

This study has three main findings. First, there are wide disparities by income in cardiovascular risk factors and prevalence in U.S. metropolitan areas. However, these disparities vary substantially between metropolitan areas. Second, several metropolitan areas appeared repeatedly among the top ten metropolitan areas with the widest disparities. Third and last, the contextual variables we explored explained between 11 % and 62 % of the heterogeneity in disparities between metropolitan areas, depending on the outcome, even after adjusting for individual characteristics. Furthermore, we observed that metropolitan areas with higher SES had wider disparities in general.

Several studies have demonstrated health disparities by income (Powell-Wiley et al., 2022, Cainzos-Achirica et al., 2019, Mullachery et al., 2022, Sallis et al., 2011, Sallis et al., 2009, Rowhani-Rahbar et al., 2019, Liu et al., 2021), and others have shown spatial or geographic variations in the magnitude of disparities (Tabb et al., 2020, Tabb et al., 2018, Tabb et al., 2020, Tabb et al., 2022, Siegel et al., 2015). However, to our knowledge, this is the first study to specifically compare the magnitude of income disparities in cardiovascular disease risk factors between core-based (metropolitan) areas throughout the US. Montez, et al. (2014) compared the four US geographic regions and found subtle regional differences in the relationship between higher education and lower mortality (Montez and Berkman, 2014). Our study, while primarily comparing metropolitan areas, also found no obvious differences in cardiovascular risk factor disparities by income level in these same four US regions. Chetty et al. (2016) found that life expectancy for low-income individuals varied significantly across local areas within the U.S. and was heavily correlated with health behaviors, such as smoking, but not with access to medical care, physical environment factors, income inequality, or labor market conditions (Chetty et al., 2016). Schnake-Mahl, Mullachery et al. (2022) also found that income-based disparities in life expectancy vary widely by metropolitan area, finding that midwestern metropolitan areas had wider disparities (Schnake-Mahl, Mullachery et al., 2022). Our study also found variability in the magnitude of health disparities across the US when comparing metropolitan areas, but we did not find a clear geographical pattern.

We also studied how heterogeneity in disparities by income was explained by individual (age, sex, race/ethnicity) and area-level (both contextual and compositional) factors. The metropolitan-level factors that we studied accounted for low amounts of heterogeneity in some outcomes (hypertension, diabetes, and cardiovascular disease), and most of the heterogeneity in others (sedentary lifestyle, smoking, and obesity). Interestingly, we found that metropolitan areas with higher SES had generally wider disparities. This is consistent with the findings of Schnake-Mahl, Jahn et al. (2022) that found wider income-based disparities in census tract life expectancy in metropolitan areas with higher SES (Schnake-Mahl, Mullachery et al., 2022), and the findings of De Ramos et al. (2022) that found wider ethnic COVID-19 inequities in cities of lower social vulnerability. (De Ramos et al., 2022) We also found that the density of PCPs in a metropolitan area was associated with wider disparities. A study of European countries by McKinnon et al. (2016) similarly found that a higher density of physicians was correlated with greater disparities in health care, though better care overall. (McKinnon et al., 2016) The authors theorized this was likely due to unequal distribution of physicians.

Our findings regarding the heterogeneity of income-related disparities in CVD risk factors among US metropolitan areas indicates a need to better understand the association between income and CVD and its risk factors. However, we caution that our analysis was conducted using metropolitan areas, which are aggregations of counties with strong commuting links with a core city. This city definition is useful in that it takes into consideration both core urban zones and surrounding suburban areas (Schnake-Mahl, Jahn et al., 2022b). Suburban populations are likely demographically distinct from urban populations, and have distinct patterns of health outcomes compared to urban populations (Schnake-Mahl and Sommers, 2017). However, metropolitan areas do not have political representation or governing bodies per se, except for metropolitan planning organizations and some metropolitan transit systems (Schnake-Mahl, Jahn et al., 2022b). A comparison of disparities within cities may have more direct policy implications, as there is an accountable governing body for cities (Schnake-Mahl, Jahn et al., 2022b), but data on city of residence is unavailable in BRFSS.

Our study has other limitations. First, our measure of income was crude, including a large upper category ($75,000 or above), but the existing categorizations of income at BRFSS precluded further disaggregation. However, these limitations of BRFSS should be weighted against its large sample size and geographic coverage. Moreover, studies have shown a logarithmic relationship between income and life expectancy. Chetty et al. demonstrated that life expectancy exhibits diminishing returns, with the most significant variation observed in lower income brackets, especially below $74,000. (Chetty et al., 2016) Future studies may benefit from use of datasets with finer income gradations, or from revisions to the current categories in BRFSS. Second, our analysis was constrained by several confounding variables related to income that could not be controlled for due again to limitations of the BRFSS dataset. The most notable of which is number of people per household, which may profoundly affect our income brackets. Those with smaller households are likely in a different socioeconomic position than those with larger households making the same income. Mills et al. (2020) noted these same limitations of the BRFSS in their study of income disparities related to smoking. (Mills et al., 2020) Third, the prevalence of diabetes, hypertension, and cardiovascular disease was assessed based on participant-reported data, which is susceptible to information bias. Additionally, diagnosis of these conditions requires access to healthcare, which may not be available to all participants. Notably however, Schneider et al. demonstrated that self-reported diabetes diagnosis (using a method similar to our study) showed over 92 % reliability over time. However, it is estimated that approximately one third of diabetes cases in the U.S. remain undiagnosed. (Schneider et al., 2012) Future studies may mitigate these issues by connecting participant surveys with electronic health record data. Fourth, we were unable to explore absolute disparities (e.g., the slope index of inequality) as Poisson multilevel additive models did not converge. Computing absolute disparities concurrently with relative disparities allow for a more complete picture of disparities (Schnake-Mahl, Mullachery et al., 2022). Fifth, we did not intend to infer causality from our findings, and these should be interpreted as descriptive results of where income-based disparities are widest. Finally, we used data from 2012 to 2019, so our results may not be generalizable to the years of the COVID-19 pandemic.

## 5 Conclusion

In this study of cardiovascular disease disparities by income, we found large heterogeneities between metropolitan areas in the U.S. Compositional and contextual variables of each metropolitan area that we studied explained most of this heterogeneity. These findings are important in better understanding the association between income inequality and CVD and its risk factors.

## CRediT authorship contribution statement

**John Kundrick:** Writing – review & editing, Writing – original draft, Visualization, Investigation, Conceptualization. **Heather Rollins:** Writing – review & editing, Visualization, Software, Resources, Methodology, Formal analysis, Data curation. **Pricila Mullachery:** Writing – review & editing, Methodology, Investigation. **Asma Sharaf:** Writing – review & editing, Visualization, Formal analysis, Data curation. **Alina Schnake-Mahl:** Writing – review & editing, Methodology, Investigation. **Ana V. Diez Roux:** Writing – review & editing, Methodology, Investigation, Conceptualization. **Usama Bilal:** Writing – review & editing, Writing – original draft, Visualization, Validation, Supervision, Software, Resources, Project administration, Methodology, Investigation, Funding acquisition, Formal analysis, Data curation, Conceptualization.

## Declaration of competing interest

The authors declare that they have no known competing financial interests or personal relationships that could have appeared to influence the work reported in this paper.

## Data Availability

Data is public
