# Supplementary material for: Heterogeneity in disparities by income in cardiovascular risk factors across 209 US metropolitan areas
Source: Prev Med Rep. 2024 Oct 19;47:102908. doi: 10.1016/j.pmedr.2024.102908 (PMC11541419; doi:10.1016/j.pmedr.2024.102908)
Supplement: Supplementary Data 1 [file mmc1.docx]

**Appendix 1: modeling strategy**

We used weighted robust multilevel Poisson models of individuals nested in metropolitan areas to estimate the magnitude of disparities for each of the six binary risk factors. We chose to employ robust Poisson models rather than logistic models due to the robust Poisson model’s lower susceptibility to biased estimates of risk ratios when the model is misspecified^1^, and to address the sensitivity of odds ratios to the baseline prevalence (which differs by metropolitan area and risk factor).

The main fixed effect exposure was individual income, and the individual-level covariates, age (continuous), sex (binary), and race/ethnicity (categorical), were added iteratively to the model. As individuals were clustered in metropolitan areas, metropolitan-level random intercept and slope for income were also included in each model. All models were also adjusted by the year the individual was sampled in, to adjust for potential changes in the income-outcome relationship over time due to effects such as inflation.

The model for a given outcome $Y$ that includes all individual level covariates for individual $i$ in metropolitan area $j$ is represented by following equation:

$$\left( 1 \right)\log y_{ij}=\beta_{0j}+\beta_{1j}{income}_{ij}+\beta_{2}{age}_{ij}+\beta_{3}{female}_{ij}+\beta_{4}{NH Black}_{ij}+\beta_{4}{Hispanic}_{ij}+\beta_{4}{NH Other}_{ij}+\beta_{5}*year_{ij}$$

$$\beta_{0j}= \gamma_{00}+u_{0j} ; \beta_{1j}= \gamma_{10}+u_{1j};$$

$$u_{0j} \sim N\left( 0,\tau_{00} \right); u_{1j} \sim N\left( 0,\tau_{11} \right); Cov\left( u_{0j},u_{1j} \right) \sim\tau_{10}$$

We rescaled the income variable into a range of 0 (highest income) to 1 (lowest income) to allow for interpretation of the $\exp\beta_{1j}$ as the relative index of inequality (RII)^2^. We assigned each income category its ridit score^3^, or the midpoint of the cumulative proportion of the population up to that category (starting with the highest category of income). For example, if the highest income category had a prevalence of 10, then we assigned it the value 0.05. If the second highest category had a prevalence of 5, then we assigned it the value of 0.075 (0.05+0.25). We computed ridit scores using both the overall (main analysis) and the metropolitan-specific income distribution. The RII summarizes the linear association between income categories and each CVD risk factor or prevalence in each metropolitan area. The fixed and random slope estimates for income, $\gamma_{10}$ and $u_{1j}$ respectively, were extracted, along with their variance-covariance estimates, to produce metropolitan-specific RII estimates with 95 confidence intervals. Robust level 1 variance-covariance estimates were calculated by using a clustered sandwich method. Summing the robust level 1 variance for income with the level 2 metropolitan-specific income variances produced robust variance terms for each metropolitan area RII for income.

To explore contextual factors driving income-related disparities, we expanded the model of the second step by adding 11 metropolitan-level variables, first individually in separate models, and then together in a full model. Each metropolitan-level variable was included in the expanded models as a main effect along with an interaction with individual level income. The interaction can be interpreted as the effect of the metropolitan area factor on the disparities by income of each outcome across metropolitan areas. For a model containing only one metropolitan-level contextual variable $X_{k}$, the terms $+\beta_{5}X_{kj}+\beta_{6}X_{kj}{income}_{ij}$ were added to the model equation. The full model containing all metropolitan-level factors $X_{k}$, $k=$ 1, …, 11, can be expressed by the level 1 equation 2 below.

$$\left( 2 \right) \log y_{ij}=\beta_{0j}+\beta_{1j}{income}_{ij}+\beta_{2}{age}_{ij}+\beta_{3}{female}_{ij}+\beta_{4}{NH Black}_{ij}+\beta_{4}{Hispanic}_{ij}+\beta_{4}{NH Other}_{ij}+\beta_{5}year_{ij}+\beta_{6}X_{1j}+\ldots+\beta_{16}X_{11j}+\beta_{17}X_{1j}{income}_{ij}+\ldots+\beta_{27}X_{11j}{income}_{ij}$$

RIIs and their robust 95 intervals were also extracted for the 11 models each containing only one metropolitan-level contextual variable. Since the values for all metropolitan area contextual variables were centered around 0, we can define ${RII}_{mean}=\exp\gamma_{10}$ as the RII for income at the mean value of the contextual variable. Since the contextual variables had also been rescaled as 1 unit = 1 standard deviation, we can define the RII for a metropolitan area with a 1 SD higher or lower value in the contextual variable as ${RII}_{+/-1sd}=exp( \gamma_{10}+/-\beta_{6})$ where $\beta_{6}$ is the coefficient for the interaction term $X_{kj}{income}_{ij}$.

Finally, we assessed the change in variability in the income disparities across metropolitan areas through the successive model iterations with additional covariates described above. We calculated the proportional change in variance (PCV) between the empty model and each adjusted model, summarized by the level 2 variance terms $\tau_{11}$, using equation 4. We hypothesized that adjusting the model with individual level and contextual variables would reduce the variability of the income random slope across metropolitan areas.

$$\left( 3 \right) PCV=\frac{\tau_{11empty}-\tau_{11adjusted}}{\tau_{11empty}} .$$

**Appendix References:**

1. Zou G. A modified poisson regression approach to prospective studies with binary data. *American journal of epidemiology*. 2004;159:702-706. doi: 10.1093/aje/kwh090

2. Moreno-Betancur M, Latouche A, Menvielle G, Kunst AE, Rey G. Relative index of inequality and slope index of inequality: a structured regression framework for estimation. *Epidemiology*. 2015;26:518-527.

3. Strand BH, Steingrímsdóttir ÓA, Grøholt E-K, Ariansen I, Graff-Iversen S, Næss Ø. Trends in educational inequalities in cause specific mortality in Norway from 1960 to 2010: a turning point for educational inequalities in cause specific mortality of Norwegian men after the millennium? *BMC Public Health*. 2014;14:1208. doi: 10.1186/1471-2458-14-1208

**Appendix Table 1: Variation of relative indices of inequality by outcome and census region, adjusted by age and sex for 209 metropolitan areas in the United States from 2012 to 2019.**

|  | **Median Relative Index of Inequality (RII) [Q1 - Q3]** | | | | | **Coefficient of Variation of RII (%)** | | | | |
| --- | --- | --- | --- | --- | --- | --- | --- | --- | --- | --- |
|  | **US** | **Northeast** | **South** | **Midwest** | **West** | **US** | **Northeast** | **South** | **Midwest** | **West** |
| **Obesity** | 1.41 [1.32 - 1.50] | 1.44 [1.37 - 1.52] | 1.39 [1.30 - 1.52] | 1.36 [1.27 - 1.44] | 1.44 [1.36 - 1.53] | 10.5 | 10.1 | 11.3 | 9.2 | 9.7 |
| **Diabetes Mellitus** | 2.94 [2.80 - 3.12] | 3.04 [2.93 - 3.15] | 2.87 [2.73 - 3.06] | 2.93 [2.81 - 3.09] | 2.99 [2.82 - 3.12] | 8.6 | 8.2 | 9.7 | 7.6 | 6.9 |
| **Smoking** | 5.27 [4.17 - 6.19] | 5.68 [4.68 - 6.45] | 4.76 [3.97 - 5.57] | 5.78 [4.80 - 6.67] | 4.87 [4.27 - 5.81] | 28.5 | 27.2 | 29.6 | 22.4 | 30.6 |
| **Sedentary Lifestyle** | 3.75 [3.35 - 4.21] | 4.11 [3.70 - 4.36] | 3.56 [3.29 - 4.11] | 3.50 [3.13 - 3.88] | 4.04 [3.50 - 4.22] | 15.7 | 11.2 | 15.7 | 16.4 | 15.1 |
| **Hypertension** | 1.53 [1.49 - 1.57] | 1.54 [1.52 - 1.56] | 1.57 [1.53 - 1.61] | 1.51 [1.48 - 1.53] | 1.48 [1.47 - 1.51] | 4.1 | 2.4 | 4.0 | 3.6 | 2.6 |
| **Cardiovascular Disease** | 3.90 [3.62 - 4.14] | 3.88 [3.64 - 4.00] | 4.12 [3.85 - 4.40] | 3.91 [3.68 - 4.19] | 3.63 [3.52 - 3.87] | 11.3 | 7.4 | 11.2 | 11.4 | 9.1 |

**Footnote: US=United States.**

**Appendix Table 2: P-Values for the Null Hypothesis of No Interaction Between each metropolitan variable and Income, for each outcome, for 209 metropolitan areas in the United States from 2012 to 2019.**

|  | **Obesity** | **Diabetes** | **Smoking** | **Sedentary** | **Hypertension** | **CVD** |
| --- | --- | --- | --- | --- | --- | --- |
| **Age <18** | 0.04 | 0.19 | 0.50 | 0.02 | 0.13 | 0.26 |
| **Age 65+** | <0.01 | 0.34 | 0.37 | 0.05 | 0.67 | 0.09 |
| **Non-Hispanic Black** | <0.01 | <0.01 | <0.01 | 0.15 | 0.15 | 0.08 |
| **Hispanic** | 0.92 | 0.54 | <0.01 | 0.81 | 0.05 | 0.02 |
| **Foreign-born** | 0.01 | 0.49 | <0.01 | 0.18 | 0.41 | <0.01 |
| **College Educ.** | <0.01 | 0.08 | 0.12 | <0.01 | 0.04 | 0.44 |
| **Poverty** | 0.04 | 0.04 | <0.01 | 0.01 | 0.21 | 0.71 |
| **Gini** | 0.18 | 0.17 | <0.01 | 0.94 | 0.50 | 0.34 |
| **Uninsured** | 0.62 | 0.12 | <0.01 | 0.03 | 0.02 | 0.70 |
| **Primary Care Physicians per capita** | <0.01 | 0.04 | <0.01 | <0.01 | <0.01 | 0.95 |
| **Unemployed** | 0.67 | 0.09 | 0.02 | 0.97 | 0.13 | 0.34 |

**Appendix Figure 1: Relative Index of Inequality and 95 Confidence Interval for all 209 metropolitan areas in the United States from 2012 to 2019, adjusted by age and sex**

Footnote: each unit is a metropolitan area, sorted in the x-axis by the relative index of inequality.

**Appendix Figure 2: Correlation between the relative indices of inequality across cardiovascular risk factors and prevalence for 209 metropolitan areas in the United States from 2012 to 2019.**

**Footnote: Corr: correlation**

**Appendix Figure 3: Comparison between relative indices of inequality when using global vs metropolitan-specific income distributions to calculate ridit scores for 209 metropolitan areas in the United States from 2012 to 2019.**

**Appendix Figure 4: Correlation between metropolitan-level variables used in this study for 209 metropolitan areas in the United States from 2012 to 2019.**

**
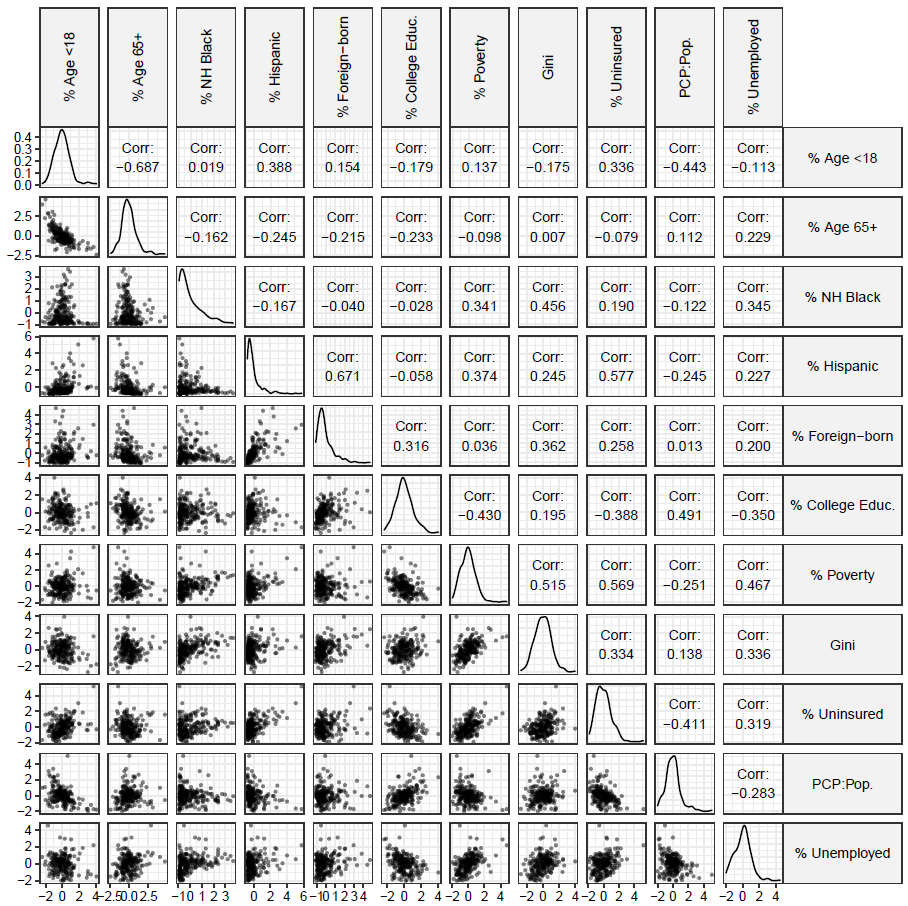
**

**Footnote: NH=non-Hispanic, PCP:pop=primary care physicians per capita**
